# Examining the Food Retail Choice Context in Urban Food Deserts, Ohio, 2015

**DOI:** 10.5888/pcd14.160408

**Published:** 2017-10-05

**Authors:** Stephanie N. Pike, Erika S. Trapl, Jill K. Clark, Chaturia D. Rouse, Bethany A. Bell, Ashwini R. Sehgal, Thomas To, Elaine Borawski, Darcy A. Freedman

**Affiliations:** 1 Department of Population and Quantitative Health Sciences, Case Western Reserve University, School of Medicine, Cleveland, Ohio; 2 Prevention Research Center for Healthy Neighborhoods, Case Western Reserve University, Cleveland, Ohio; 3 John Glenn College of Public Affairs and Food Innovation Center, Ohio State University, Columbus, Ohio; 4 College of Social Work, University of South Carolina, Columbia, South Carolina; 5 Center for Reducing Health Disparities, Case Western Reserve University, Cleveland, Ohio; 6 School of Health Sciences, Cleveland State University, Cleveland, Ohio

**Figure:**
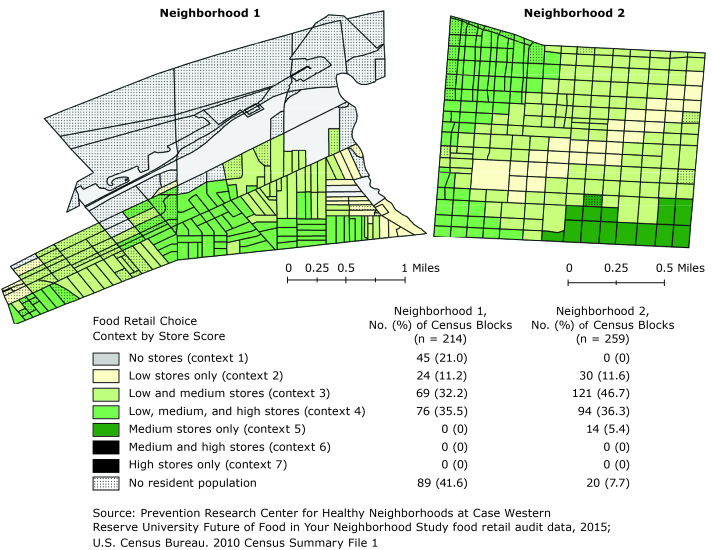
Distribution of food retail choice contexts within 2 urban food desert neighborhoods, Ohio, 2015. Store scores (low, ≤10; medium, 11–29; high, ≥30) are based on Nutrition Environment Measures Survey in Convenience Stores (NEMS-CS) and Bridging the Gap Community Obesity Measures Project (BTG-COMP).

## Background

The US Department of Agriculture (USDA) characterizes food deserts as low-income neighborhoods that distinctly lack supermarkets and grocery stores (1). This definition elevates the importance of large food retailers where Americans spend most of their food dollars and deemphasizes the contributions of smaller food stores such as convenience and dollar stores for food choice decision making. Smaller food retailers are more prevalent than large food retailers (2), and excluding them from the conceptualization of food deserts has implications for research, policy, and practice focused on reducing chronic disease through improvements to local food environments.

Food deserts are associated with chronic conditions including obesity, heart disease, and diabetes, but the association is not fully explained by the existence or absence of a large food retailer (3). Even when these retailers are present, the prevalence of obesity is significantly higher if convenience stores also are present (4). Furthermore, introducing a new supermarket in a neighborhood has had mixed effects on dietary behaviors (5). This evidence suggests that, although physical access to large food retailers is important, the environmental factors that shape dietary choice are far more complex.

We sought to systematically evaluate the food retail choice context in 2 urban neighborhoods that are USDA-designated food deserts because of lack of access to large food retailers within one-half mile of most census tracts (composed of census blocks). Our aim was to develop a method for evaluating variability in the food retail choice context by examining availability, pricing, quality, and advertising of healthy food items among all food retailers in these neighborhoods.

## Methods

We evaluated every food retailer in 2 racially and economically matched neighborhoods from 2 metropolitan areas in Ohio. In the targeted neighborhoods, more than 40% of the population lived below the federal poverty level, and more than 70% identified as a racial/ethnic minority (6). We observed all food retail outlet types including convenience stores, gas stations, pharmacies, dollar stores, and ethnic and specialty food stores located in and on the periphery (ie, directly across the street) of neighborhood boundaries. The nearest supermarket to each neighborhood commonly used by residents was included to account for the reality that these stores are part of the food retail choice context for residents who cross neighborhood boundaries to access a large food retailer.

Each store was audited independently by 2 trained researchers using an adapted Nutrition Environment Measures Survey in Convenience Stores (NEMS-CS), a standardized tool for evaluating availability, price, and quality of healthy food options among 10 different food categories: milk, fruits and vegetables (fresh, frozen, canned), ground beef, hot dogs, frozen dinners, baked goods, beverages, bread, chips, and cereal (7). To evaluate healthy and unhealthy advertising on store exteriors, we used an adapted Food Store Observation Form from the Bridging the Gap Community Obesity Measures Project (BTG-COMP) (8). Using both the NEMS-CS and the BTG-COMP tools, a score for each store was calculated; possible scores ranged from −13 to 65. Stores were categorized into 3 groups on the basis of literature on how grocery and convenience stores have been scored using similar NEMS measures (7,9). Score categories were low (≤10), medium (11–29), and high (≥30); lower scores are associated with lower availability, higher pricing, and reduced quality of healthy food options as well as higher rates of unhealthy food or product advertising.

Store addresses were geocoded using ESRI ArcMap 10.3 (ESRI) and overlaid with a one-half mile network buffer based on adapted USDA methodology for determining food desert status. Next, food retail choice context scores for census blocks (n = 473 blocks) were calculated by counting the number of low, medium, and high scoring NEMS/BTG-COMP stores whose one-half mile buffer intersected the centroid of the census block. Using this methodology, there were 7 possible food retail choice context categories that could be observed: no stores (choice context 1), low-scoring stores only (choice context 2), low- and medium-scoring stores (choice context 3), low-, medium-, and high-scoring stores (choice context 4), medium-scoring stores only (choice context 5), medium- and high-scoring stores (choice context 6), and high-scoring stores only (choice context 7). 

## Main Findings

The average scores for stores (n = 55) in the 2 neighborhoods using the combined NEMS-CS/BTG-COMP measure was 6.7 for the low score category, 18.8 for the medium score category, and 41.3 for the high score category (Table). Most stores (63.6%) scored in the low category; only 7.3% scored in the high category. Five of the 7 food retail choice contexts were observed; choice contexts 6 and 7 were not observed. In Neighborhood 1, 21.0% of blocks had no stores (choice context 1) but many of these blocks had no resident population. In Neighborhood 2, nearly half of all blocks (46.7%) had a mixture of low score and medium score stores (choice context 3). Census blocks with medium scoring stores only (choice context 5) were minimally present in Neighborhood 2 (5.4%) and absent from Neighborhood 1.

**Table:** Scoring of Availability, Price, Quality, and Advertising of Healthy Foods Among All Food Retailers in Urban Food Desert Neighborhoods, Ohio, 2015

Characteristic	Low^a^ Score Stores (n = 35)	Medium^a^ Score Stores (n = 16)	High^a^ Score Stores (n = 4)	All Stores (n = 55)
**NEMS-CS/BTG-COMP score, mean (SD)**				
Availability^b^	6.3 (3.1)	12.8 (3.3)	29.5 (5.5)	9.9 (7.1)
Price^c^	1.3 (1.7)	4.3 (2.2)	5.5 (2.6)	2.5 (2.5)
Quality^d^	0.2 (1.0)	2.6 (2.7)	6.0 (0)	1.3 (2.4)
Advertising^e^	−1.2 (1.2)	−0.9 (1.1)	0.3 (0.5)	−1.0 (1.2)
Total^f^	6.7 (4.1)	18.8 (4.3)	41.3 (6.4)	12.7 (10.6)
**Available healthy food options, no. (%)**				
Skim, 1%, or 2% milk	18 (51)	11 (73)	4 (100)	33 (60)
Fresh fruit	3 (9)	8 (50)	4 (100)	15 (27)
Canned fruit	16 (46)	13 (81)	4 (100)	33 (60)
Fresh vegetables	1 (3)	5 (32)	4 (100)	10 (18)
Canned vegetables	27 (77)	16 (100)	4 (100)	47 (86)
Lean ground beef (<10% fat per pound)	1 (3)	2 (13)	3 (75)	6 (11)
Lean hot dogs (≤9 g fat per serving)	2 (6)	10 (63)	4 (100)	16 (29)
Low-fat frozen dinners (≤9 g fat per serving)	4 (11)	7 (44)	4 (100)	15 (27)
Low-fat baked goods (≤3 g fat per serving)	0	1 (6)	4 (100)	5 (9)
Diet soda	31 (89)	14 (88)	4 (100)	49 (89)
100% juice	29 (83)	14 (88)	3 (75)	46 (84)
Whole wheat bread	0	3 (19)	4 (100)	7 (13)
Baked chips (≤3 g fat per serving)	4 (11)	3 (19)	2 (50)	9 (16)
Low-sugar cereal (<7 g sugar per serving)	13 (37)	11 (69)	4 (100)	28 (51)

Abbreviations: BTG-COMP, Bridging the Gap Community Obesity Measures Project; NEMS-CS, Nutrition Environment Measures Survey in Convenience Stores; SD, standard deviation.

a Score categories low (≤10), medium (11–29), and high (≥30) are based on total NEMS-CS/BTG-COMP scores; lower scores are associated with lower availability, higher pricing, and reduced quality of healthy food options and higher rates of advertising of unhealthy foods.

b Possible availability scores ranged from 0 to 38. Stores gained points for having healthy items in 10 categories: milk, fruits and vegetables (fresh, frozen, canned), ground beef, hot dogs, frozen dinners, baked goods, beverages, bread, chips, and cereal.

c Possible pricing scores ranged from −9 to 18. Stores lost points for having healthy items that were more expensive than the unhealthy alternative.

d Possible quality scores ranged from 0 to 6. Quality was observed for fresh fruit and vegetables.

e Possible advertising scores ranged from −4 to 3. Stores lost points for having more than 50% of total advertisement for unhealthy foods or products (eg, tobacco and alcohol advertisements).

f Total possible score ranged from −13 to 65.

The most common healthy options available among all stores were canned vegetables, 100% juice, and diet soda (Table). None of the stores categorized as low sold whole-wheat bread or low-fat baked goods, and fewer than 10% of these stores sold fresh fruits or vegetables, lean ground beef, or lean hot dogs. Although stores categorized as medium were more likely to carry items among each of the 10 food categories, fewer than one-third sold fresh vegetables, lean ground beef, low-fat baked goods, whole-wheat bread, or baked chips. Stores categorized as high had at least 1 healthy item available in each food category.

Three-fourths (74.5%) of stores had advertisements on the building exterior or property, with a mean of 17.5 advertisements per store. For 92.3% of stores, more than half of the advertisements were for tobacco or alcohol products. Advertisements for sugar-sweetened beverages (ie, energy drinks, soda) were found among 39% of stores; 7.3% had an advertisement for a sugarless drink product (ie, diet soda). Advertisements for a health-related behavior such as a flu shot, health insurance, or hypertension prevention were found on 5.5% of the stores.

## Action

This research describes a new method for measuring food retail choice contexts within neighborhoods. Findings suggest there is heterogeneity in food retail choice in urban food deserts; however, overall healthy food access remains limited. Methods that emphasize large food retailers within definitions of food deserts or smaller food retailers within definitions of food swamps (ie, places in which unhealthy foods are more available than healthy foods) do not capture the synergy of these stores within local food environments, which combine to shape dietary decision-making (10). Measures of food retail choice contexts may provide a more precise indication of how and where to target future food environment interventions.

## References

[1] US Department of Agriculture. Food access research atlas: documentation. Washington (DC): Economic Research Service. https://www.ers.usda.gov/data-products/food-access-research-atlas/documentation/#definitions. Accessed March 9, 2017.

[2] Scope of the industry: convenience stores offer more convenience. Alexandria (VA): The Association for Convenience and Fuel Retailing. http://www.nacsonline.com/Research/FactSheets/ScopeofIndustry/Pages/Convenience.aspx. Accessed December 9, 2015.

[3] Beaulac J , Kristjansson E , Cummins S . A systematic review of food deserts, 1966–2007. Prev Chronic Dis 2009;6(3):A105. 19527577 PMC2722409

[4] Pearce J , Hiscock R , Blakely T , Witten K . The contextual effects of neighbourhood access to supermarkets and convenience stores on individual fruit and vegetable consumption. J Epidemiol Community Health 2008;62(3):198–201. 10.1136/jech.2006.059196 18272733

[5] Dubowitz T , Ghosh-Dastidar M , Cohen DA , Beckman R , Steiner ED , Hunter GP , Diet and perceptions change with supermarket introduction in a food desert, but not because of supermarket use. Health Aff (Millwood) 2015;34(11):1858–68. 10.1377/hlthaff.2015.0667 26526243 PMC4977027

[6] US Census Bureau. S1703: Select characteristics of people at specified levels of poverty in the past 12 months: 2009–2013 American Community Survey 5-Year Estimates. US Census Bureau’s American Community Survey Office; 2015. https://factfinder.census.gov. Accessed March 9, 2017.

[7] Cavanaugh E , Mallya G , Brensinger C , Tierney A , Glanz K . Nutrition environments in corner stores in Philadelphia. Prev Med 2013;56(2):149–51. 10.1016/j.ypmed.2012.12.007 23262362

[8] Bridging the gap community obesity measures project. Chicago (IL): University of Illinois at Chicago, Institute for Health Research and Policy; 2012. http://www.bridgingthegapresearch.org/research/community_data/. Accessed September 1, 2015.

[9] Hillier A , McLaughlin J , Cannuscio CC , Chilton M , Krasny S , Karpyn A . The impact of WIC food package changes on access to healthful food in 2 low-income urban neighborhoods. J Nutr Educ Behav 2012;44(3):210–6. 10.1016/j.jneb.2011.08.004 22405817

[10] Rose D , Bodor JN , Hutchinson PL , Swalm CM . The importance of a multi-dimensional approach for studying the links between food access and consumption. J Nutr 2010;140(6):1170–4. 10.3945/jn.109.113159 20410084 PMC2869502

